# Preeclampsia screening and prevention—A Nordic perspective

**DOI:** 10.1111/aogs.15073

**Published:** 2025-02-14

**Authors:** Charlotte K. Ekelund, Ylva Carlsson, Lina Bergman, Anna‐Karin Wikström, Kjell Å. B. Salvesen, Vedran Stefanovic, Pia M. Villa, Jóhanna Gunnarsdóttir, Line Rode

**Affiliations:** ^1^ Fetal Medicine Unit, Department of Obstetrics and Gynecology Rigshospitalet Copenhagen Denmark; ^2^ Faculty of Health Sciences University of Copenhagen Copenhagen Denmark; ^3^ Department of Obstetrics and Gynecology Sahlgrenska University Hospital Gothenburg Sweden; ^4^ Centre of Perinatal Medicine & Health, Institute of Clinical Sciences, Sahlgrenska Academy University of Gothenburg Gothenburg Sweden; ^5^ Department of Obstetrics and Gynecology, Institute of Clinical Sciences, Sahlgrenska Academy University of Gothenburg Gothenburg Sweden; ^6^ Department of Obstetrics and Gynecology Stellenbosch University Cape Town South Africa; ^7^ Department of Women's and Children's Health Uppsala University Uppsala Sweden; ^8^ Department of Clinical and Molecular Medicine, Faculty of Medicine and Health Sciences NTNU, Norwegian University of Science and Technology Trondheim Norway; ^9^ Department of Obstetrics and Gynecology Trondheim University Hospital Trondheim Norway; ^10^ Fetomaternal Medical Center, Department of Obstetrics and Gynecology Helsinki University Hospital and University of Helsinki Helsinki Finland; ^11^ Department of Obstetrics and Gynecology Helsinki University Hospital and University of Helsinki Helsinki Finland; ^12^ Faculty of Medicine University of Iceland Reykjavik Iceland; ^13^ Department of Obstetrics and Gynecology Landspitali—The National University Hospital of Iceland Reykjavik Iceland; ^14^ Department of Clinical Biochemistry Rigshospitalet Copenhagen Denmark

Preeclampsia, particularly if onset is early, possess a significant challenge for obstetricians. Despite advances in maternal healthcare, the global incidence of preeclampsia remains at 2%–8%, with Nordic countries reporting rates around 3%–4%. Delivery is the only effective treatment, resulting in high perinatal mortality and morbidity due to preterm birth. Furthermore, preeclampsia currently remains one of the leading causes of maternal mortality and severe maternal morbidity worldwide.

Over time, various preventive strategies have been explored, and while ongoing pharmaceutical studies aim to identify alternative options, a low‐dose of acetylsalicylic acid (aspirin) has, so far, proven to be the most effective. Meta‐analyses indicate that aspirin at a dose around 100 mg/day, administered to high‐risk women before 16 weeks of gestation, can reduce the risk of preterm preeclampsia (preeclampsia with delivery before 37 weeks of gestation).^1^ However, the exact dose and possible benefit if treatment is started after 16 weeks of gestation is still being explored.

The definition of ‘high‐risk’ for preeclampsia and eligibility for aspirin prophylaxis varies across national obstetric guidelines. In the Nordic countries (Denmark, Finland, Iceland, Norway and Sweden), guidelines recommend aspirin treatment for women based on maternal or obstetric risk factors. This can be either a single high‐risk factor, such as pregestational diabetes, or a history of preeclampsia. In some national guidelines women with multiple moderate‐risk factors like high body mass index and nulliparity are also eligible for aspirin prophylaxis. However, this screening approach, based solely on maternal/obstetric risk factors, has limited predictive accuracy. A Danish study found that screening by maternal high‐risk factors alone detects less than 30% of preterm preeclampsia cases,^2^ and a similar study evaluating the current Swedish guidelines showed only 20%–30% of women eventually developing preeclampsia are detected.^3^

Recently, a novel screening method using a multi‐marker algorithm proposed by the Fetal Medicine Foundation has attracted attention. This first‐trimester screening algorithm combines maternal risk factors with measurement of mean arterial pressure, uterine artery Doppler pulsatility index, and biochemical markers, such as placental growth factor (PlGF) and pregnancy‐associated plasma protein‐A (PAPP‐A). Studies indicate this method can identify up to 70%–75% of women at risk for preterm preeclampsia for a screen‐positive rate of 10%,^4^ surpassing the predictive accuracy of current guidelines using maternal and obstetric risk factors alone. Additionally, the ASPRE (Combined Multimarker Screening and Randomized Patient Treatment with Aspirin for Evidence‐Based Preeclampsia Prevention) trial demonstrated that women, identified as high‐risk using this Fetal Medicine Foundation algorithm and treated with 150 mg of aspirin daily, experienced a reduction of over 60% in preterm preeclampsia incidence.^5^

In Denmark, the PRESIDE (PREeclampsia Screening In DEnmark) study compared the current screening approach to the Fetal Medicine Foundation algorithm, finding the latter significantly more effective in a Danish population.^6^ In Sweden, the IMPACT (Improving Maternal Pregnancy And Child ouTcomes) study is currently evaluating the performance of the Fetal Medicine Foundation algorithm and an alternative model tailored to the Swedish population, with expected results during 2025.^7^ Some centers have already implemented the first‐trimester preeclampsia screening in Sweden and are now evaluating the effect retrospectively. Norway is conducting prospective local studies on different screening concepts, while Iceland is planning biomarker data collection for research. Finland is also exploring biomarker evaluations locally.

While literature increasingly supports shifting to a multi‐marker screening model followed by aspirin prophylaxis for the high‐risk group, widespread implementation requires evaluating the impact on preterm preeclampsia incidence in large clinical cohorts. Single and multiple center studies have evaluated and reported their results on the preterm preeclampsia incidence following implementation. Some show the expected decrease, and others do not find a significant change.^8^, ^9^ No national implementation studies have been published so far. Denmark plans to initiate a national initiative (PREPRED, PREeclampsia PREvention in Denmark) in 2025 (results expected in 2027), using a multiple baseline interrupted time series design to assess uptake and compliance, effect on the incidence of preterm preeclampsia, and evaluate maternal and neonatal complications following implementation of first‐trimester screening in Denmark. Establishing biochemical and ultrasound quality control standards is also part of the implementation plan, as continuous monitoring and maintenance of a quality control program is considered essential when running large screening programs. Compliance is another important but often overlooked factor which can impact the effect of prenatal screening programs, and adherence to prescribed aspirin prophylaxis also needs attention when preparing cost‐effectiveness analyses. Evaluation of cost is crucial and a prerequisite before a National Health Board is willing to evaluate a new screening program. These economic reports are often based on assumptions and strongly influenced by the existing pregnancy care provided before the proposed change, making them specific to each country. Studies worldwide have found the Fetal Medicine Foundation first‐trimester screening algorithm to be cost‐effective. In Norway, health economic analysis shows cost savings,^10^ while cost analyses are ongoing in Denmark and Sweden.

The Nordic countries, with their robust healthcare infrastructure and commitment to public health, are well‐positioned to lead this paradigm shift in maternal care. However, implementation challenges remain, including infrastructure limitations, resource constraints, and the attitudes and motivations of women regarding their engagement in the screening, as well as those of healthcare professionals. Ethical, political, and economic factors must also be considered. While each country faces unique hurdles, collaborative efforts within the Nordic region are invaluable, given the shared characteristics of their populations and healthcare systems. We hope for and plan that sharing of research ideas, data and results will be beneficial and eventually establish improved evidence‐based national policies on screening for and prevention of preeclampsia in the Nordic countries.
